# Oncogenic activation revealed by FGFR2 genetic alterations in intrahepatic cholangiocarcinomas

**DOI:** 10.1186/s13578-023-01156-7

**Published:** 2023-11-14

**Authors:** Xiaohong Pu, Liang Qi, Jia Wu Yan, Zihe Ai, Ping Wu, Fei Yang, Yao Fu, Xing Li, Min Zhang, Beicheng Sun, Shen Yue, Jun Chen

**Affiliations:** 1 Department of Pathology, Drum Tower Hospital, Affiliated Hospital of Medical School,Nanjing University, Nanjing, 210008 Jiangsu China; 2 Department of Radiology, The First Affiliated Hospital of Nanjing Medical University, Nanjing, 210008 Jiangsu China; 3 Department of Hepatobiliary Surgery, Drum Tower Hospital, Affiliated Hospital of Medical School,Nanjing University, Nanjing, 210008 Jiangsu China; 4 Department of Medical Genetics, Nanjing Medical University, Nanjing, 210008 Jiangsu China; 5 Shanghai Origimed Limited Company, Shanghai, 20000 China; 6 Beijing Gene Plus Limited Company, Beijing, 10000 China

**Keywords:** Intrahepatic cholangiocarcinoma, FGFR2, Translocation, In-frame deletion

## Abstract

**Background:**

Except for gene fusions, FGFR2 genetic alterations in intrahepatic cholangiocarcinomas (ICCs) have received limited attention, leaving patients harboring activating FGFR2 gene mutations with inadequate access to targeted therapies.

**Experimental design:**

We sought to survey FGFR2 genetic alterations in ICC and pan-cancers using fluorescence in situ hybridization and next-generation sequencing. We conducted an analysis of the clinical and pathological features of ICCs with different FGFR2 alterations, compared FGFR2 lesion spectrum through public databases and multicenter data, and performed cellular experiments to investigate the oncogenic potential of different FGFR2 mutants.

**Results:**

FGFR2 gene fusions were identified in 30 out of 474 ICC samples, while five FGFR2 genetic alterations aside from fusion were present in 290 ICCs. The tumors containing FGFR2 translocations exhibited unique features, which we designated as the “FGFR2 fusion subtypes of ICC”. Molecular analysis revealed that FGFR2 fusions were not mutually exclusive with other oncogenic driver genes/mutations, whereas FGFR2 in-frame deletions and site mutations often co-occurred with TP53 mutations. Multicenter and pan-cancer studies demonstrated that FGFR2 in-frame deletions were more prevalent in ICCs (0.62%) than in other cancers, and were not limited to the extracellular domain. We selected representative FGFR2 genetic alterations, including in-frame deletions, point mutations, and frameshift mutations, to analyze their oncogenic activity and responsiveness to targeted drugs. Cellular experiments revealed that different FGFR2 genetic alterations promoted ICC tumor growth, invasion, and metastasis but responded differently to FGFR-selective small molecule kinase inhibitors (SMKIs).

**Conclusions:**

FGFR2 oncogenic alterations have different clinicopathological features and respond differently to SMKIs.

**Supplementary Information:**

The online version contains supplementary material available at 10.1186/s13578-023-01156-7.

## Highlights

The findings presented in this study highlight the importance of identifying FGFR2 fusion subtypes and mutations in ICCs for both diagnosis and potential therapeutic interventions. The distinct characteristics observed in FGFR2 fusion subtypes, such as mass-forming histopathological type and CD56 positivity, can aid in accurate histopathological classification of ICC. The observation that different FGFR2 mutants in ICC nearly all promote tumor growth, invasion, and metastasis but respond differently to FGFR-selective small molecule kinase inhibitors (SMKIs) implies the need for personalized treatment strategies based on the specific FGFR2 mutation profile of each patient. These findings emphasize the potential clinical utility of targeted therapies that inhibit FGFR signaling pathways in ICC, but also underscore the importance of selecting the appropriate SMKI based on the specific FGFR2 mutation present. These findings may contribute to improved diagnosis, risk stratification, and development of targeted therapies tailored to the individual patient.

**Supplementary Information:**

The online version contains supplementary material available at 10.1186/s13578-023-01156-7.

## Introduction

Cholangiocarcinoma, a malignancy of the bile duct, is a highly aggressive neoplasm associated with a poor prognosis [1, 2]. It is classified into intrahepatic cholangiocarcinoma (ICC), perihilar cholangiocarcinoma (PHCC), or distal cholangiocarcinoma (DCC) according to tumor location within the biliary tree, as per the fifth World Health Organization Digestive System Tumors Classification [3]. In recent years, precision medicine treatment has emerged as a viable option for ICC patients, with the US FDA approving pemigatinib, a second-line ATP-competitive FGFR kinase inhibitor, for the treatment of advanced cholangiocarcinoma specifically featuring fibroblast growth factor receptor 2 (FGFR2) translocation [4].

The full-length FGFR2 protein consists of an extracellular region, three immunoglobulin (Ig)-like domains, a single hydrophobic transmembrane segment and two cytoplasmic tyrosine kinase (TK) domains [5]. FGFR2 translocation is a frequent event in ICC, observed in approximately 15% of cases [6–12]. Diverse fusion partners activating FGFR2-mediated signaling pathways have been identified, leading to receptor dimerization and downstream signaling pathway activation [13, 14]. The kinase domains and extracellular dimerization or oligomerization domains at the C-terminus are often retained in active FGFR2 fusion proteins [13]. In addition to FGFR2 translocations, site mutations, extracellular domain in-frame deletions, and truncated mutations have been reported, which may also activate the kinase domain, thus representing promising targets for precision medicine treatment [15–17]. It is unclear whether all FGFR2 genetic alterations confer oncogenic activity in ICC and elicit a response to FGFR-selective small molecule kinase inhibitors (SMKIs). The prevalence and clinicopathological features of FGFR2 genetic alterations in ICC are not yet fully understood.

The present study aimed to assess the spectrum of FGFR2 genetic lesions in 474 ICC patients using fluorescent in situ hybridization (FISH) and next-generation sequencing (NGS). Furthermore, we analyzed FGFR2 site mutations and in-frame deletions in a pan-cancer approach using NGS. By characterizing the distinct FGFR2 genetic alterations in multiple centers and pan-cancer studies, we aimed to determine the prevalence of different FGFR2 genetic alterations in our population. Additionally, we aimed to establish an effective algorithm for facilitating further genetic testing by summarizing the clinicopathological and morphological features of the various FGFR2 genetic alterations. Importantly, we evaluated the oncogenic potential and SMKI sensitivity of diverse FGFR2 genetic alterations in human intrahepatic biliary epithelial and mouse normal hepatical cell lines.

## Methods

### Patient data

**Fig. 1:**
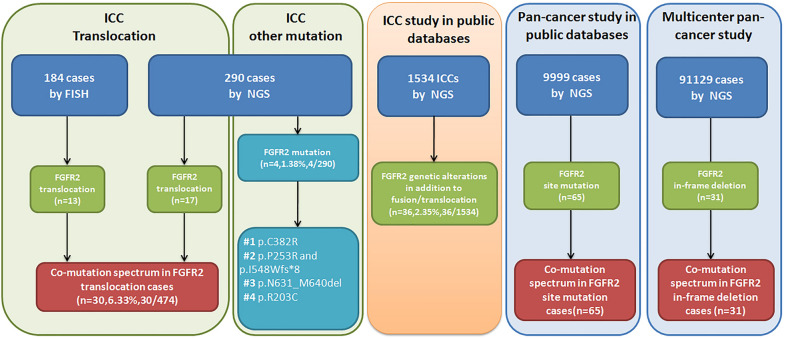
Study design. 474 ICCs were included in this study. Among 474 cases, 290 ICCs were analyzed both by FISH and NGS testing (DNA-based and RNA-based) for FGFR2 genetic alterations, and the remaining 184 cases were only analyzed by FISH. In total, there were thirty patients with FGFR2 fusion/translocation and four patients with FGFR2 genetic mutations in our cohort. Further, we selected 1534 ICC patients in 10 studies from public databases and found 38 FGFR2 genetic alterations excluding fusions in 36 patients. In addition, we also collected 31 cases with FGFR2 in-frame deletions and 65 cases with FGFR2 site mutations across multiple cancers. Finally, we depicted the comutation plot of these FGFR2 translocation/fusion, in-frame deletion and site mutation cases

We established a selected cohort of intrahepatic cholangiocarcinoma (ICC) patients undergoing surgical resection by retrieving all cases from January 2004 to December 2022 in the computerized database of the Department of Pathology, Nanjing Drum Tower Hospital, Nanjing, China. Patient consent for surgical resection and clinical research was obtained in all cases before surgical resection. The Medical Ethics Committee gave ethics approval for this study at Nanjing Drum Tower Hospital. Finally, 474 ICCs were included in this study and analyzed by FGFR2 break-apart FISH testing. Among 474 cases, 290 ICCs were also analyzed both by FISH and NGS testing (DNA-based and RNA-based) for FGFR2 genetic alterations, and the remaining 184 cases were only analyzed by FISH. In total, there were thirty patients with FGFR2 fusion/translocation and four patients with FGFR2 genetic mutations in our cohort. In addition, we analyzed 1534 ICC patients in 10 studies from public databases (https://www.cbioportal.org) to analyze FGFR2 genetic alterations (except for translocation). We also collected 91,129 cases to clarify the prevalence of FGFR2 in-frame deletions and search for FGFR2 site mutations in 9999 cases from a public database (https://www.cbioportal.org/study/summary-id=pan_origimed_2020) across multiple cancers to depicted a computation plot of these cases. The whole study design is shown in Fig. 1.

### FISH

Four-micrometer-thick, formalin-fixed and paraffin-embedded tissue sections were used for FISH. FISH testing for FGFR2 gene rearrangements was performed using the FGFR2 Dual Color Break Apart Probe (Anbiping, China). FGFR2 gene break-apart was performed and interpreted according to a previously described method [18].

### Immunohistochemistry (IHC)

CD56 (Clone: UMAB83, dilution 1:150, ZSGB-BIO, China), MUC5AC (Clone: MRQ-19, dilution 1:200, ZSGB-BIO, China), MUC6 (Clone: MRQ-20, dilution 1:200, ZSGB-BIO, China) IHC staining was carried out on an automatic Ventana Bench Mark Ultra system (Roche Diagnostics, Basel, Switzerland) using an automated staining protocol.

All IHC staining scores were calculated by multiplying the staining intensity (0 = no staining, 1 = mild staining, 2 = moderate staining, and 3 = strong staining) by the percentage of immunoreactive tumor cells (0 to 100). The immunostaining result was considered to be 0 or negative when the score was < 25; 1 + or weak when the score was 26–100; 2 + or moderate when the score was 101–200; or 3 + or strong when the score was 201–300. The IHC results were interpreted independently by two pathologists who were blinded to all clinical and pathological data.

### DNA and RNA-based next-generation sequencing

DNA and RNA from tumor tissues were extracted, and sequencing libraries were prepared. DNA-based target sequencing was performed on a panel of 1021 cancer-related genes. Complete DNA and RNA sequencing was performed on a Gene + Seq 2000 (Beijing Gene Plus, Beijing, China.) or DNBSEQ-T7 (Beijing Genomics Institute, Beijing, China.) instrument. Sequencing data were analyzed using the default parameters. The reads with removed adaptor sequences and low-quality reads were aligned to the reference human genome (hg19) using the Burrows‒Wheeler Aligner (BWA; version 0.7.12-r1039). Realignment and recalibration were performed by using GATK (version 3.4-46-gbc02625). Single nucleotide variants (SNVs) were called using MuTect (version 1.1.4) and NChot, software developed in-house to review hotspot variants. Small insertions and deletions (Indel) were determined using GATK. Somatic copy number variations (CNVs) were identified using CONTRA (v2.0.8). The fusion genes were identified with the NCsv program (in-house) using split reads, discordant pair reads, and single unmapped reads in the alignment file. The final candidate variants were all manually verified using the Integrative Genomics Viewer.

### Construction of recombinant lentivirus expressing FGFR2 mutants

cDNAs of full-length FGFR2 genetic alterations (FGFR2 p.H167-N173del, p.I288-D304del, p.N631-M640del, p.K545del, p.C382R,p.I548Wfs*8 and FGFR2-BICC1 fusion) were isolated from the corresponding tumor specimens by RT-PCR using PrimeSTAR GXL polymerase (Takara Bio) and specific primers. Each cDNA was subcloned into a pRK5 vector containing an N-terminal Flag tag (Cell Biolabs, San Diego, CA) using a homologous recombination kit (ClonExpress^®^ Entry, Nanjing, China) to generate a plasmid expressing the mutants. Each cDNA was subcloned into a GLV2-CMV-EGFP-MCS-PGK-Puro vector (Cell Biolabs, San Diego, CA) to generate recombinant lentivirus expressing the FGFR2 mutants with a FLAG epitope tag.

### Cell culture

Human cholangiocarcinoma cell lines RBE and human intrahepatic biliary epithelial cell lines HIBEC were cultured in RPMI 1640 (WISENT INC.) medium supplemented with 10% heat-inactivated fetal bovine serum (FBS) (Gibco), 1% penicillin/streptomycin (Gibco). Normal mouse hepatical cell lines (AML12) and mouse NIH3T3 fibroblast cells were maintained in DMEM with 10% FBS. Cells were grown as monolayer cultures and maintained in a humidified atmosphere with 5% CO_2_ at 37 °C.

### Proliferation assays

Cells were infected with lentivirus carrying indicated different FGFR2 mutations. Exponentially growing cells were separately seeded in 96-well plates at a density of 1 × 10^4^ cells/well. After adhesion, cell growth was evaluated by CCK8 assay (#K1018, APExBIO, USA) for 0–96 h. After incubation with CCK8 (1:10) for 2 h, cells were counted by reading the absorbance at 450 nm using a Microplate reader (SpectraMaxiD5, Molecular Devices, USA). Each sample had at least three duplicate wells and was independently performed in triplicate. The following formulae were used for calculations: Relative cell proliferation (Folds) = ([OD (experiment)–OD (blank)])/([OD (0 h)–OD (blank)]).

### Immunoblot analysis

Transfected NIH3T3 cells were lysed in RIPA buffer for Western analysis. The primary antibodies were antibodies against FLAG tag (#F1804, Sigma, USA).

### Transwell migration assay

HIBEC, AML12 and NIH3T3 cells grown in 6-well plates were transfected with different FGFR2 mutants. Transwell migration assay was performed using transwell inserts (MCEP24H48, Millipore) with a filter of 8 μm pore. A total of 2.5 × 10^4^ cells in serum-free medium were seeded into the upper chamber of the insert and complete medium was added to the lower chamber. After 24 h incubation, the cells were fixed with 4% PFA and stained with crystal violet. Then cells on the top surface of the membrane were wiped off, and cells on the lower surface were examined with a microscope at 100× magnification. Four random fields were photographed for counting and the average number of migrated cells was used as a measure of migration capacity.

### Cell viability assay

RBEwere infected with recombinant lentiviruses expressing different FGFR2 fusion mutants, then were distributed into 96-well plates with indicated concentrations of BGJ398 (#T1975, TargetMol, USA). After 72 h treatment, cell viability was evaluated by CCK8 assay as described above.

### Phalloidin cytoskeleton staining

Transfected cells were seeded on glass coverslips for 24 h and fixed with 4% PFA for 20 min at 4 ℃, then the cells were permeabilized with 0.5% TritonX-100 for 10 min at room temperature. Fluorescently labeled phalloidin working solution was added a well and incubated at room temperature for 30 min for staining and stained with DAPI. The cell cytoskeleton images were acquired with laser scanning confocal microscope.

### Statistical analysis

All data analyses were performed using SPSS 19.0 software (SPSS Inc., Chicago, Illinois, US). Fisher’s exact test was used for categorical data, and Student’s t test was used for continuous data. Analysis of variance or the Kruskal‒Wallis rank sum test was used to compare differences among different groups. The chi-square or Fisher's exact test was utilized for comparison of ratios. Differences were considered to be statistically significant when p values were less than 0.05.

## Results

### FGFR2 lesion spectrum in ICC and pan-cancer

We collected paraffin blocks of resected ICCs from 474 cases in our hospital between 2004 and 2022. To investigate the genetic features of these ICCs, FISH was conducted using the FGFR2 Dual Color Break Apart Probes (Additional file 1: Fig. S1) to test FGFR2 gene rearrangements in all tumor samples [18]. Furthermore, a subgroup of 290 tumors was subjected to whole genome-wide transcriptomic RNA sequencing and genomic DNA sequencing using next-generation sequencing (NGS) as two separate projects (Fig. 1). In all, we uncovered 30 cases carrying FGFR2 translocations (6.33%, 30/474) by the combination of FISH and sequencing efforts. Additional file 7: Table S1 presents the clinicopathologic and molecular characteristics, and follow-up data for patients who displayed FGFR2 fusion/translocation.

**Fig. 2:**
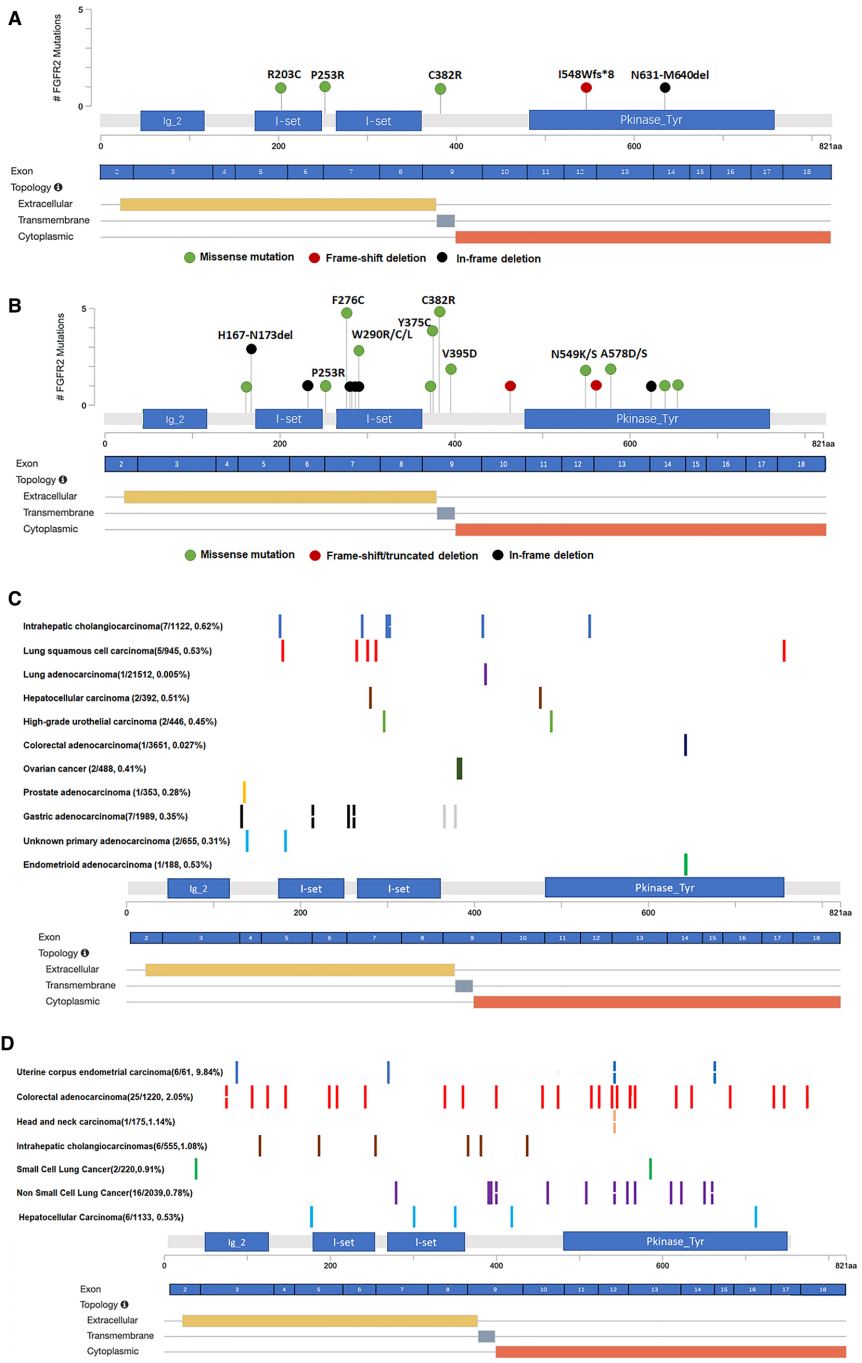
FGFR2 lesion spectrum in a large cohort of ICC and pan-cancer patients. In a total of 290 ICCs, five FGFR2 genetic alterations (except for translocation/fusion) were found, the types of genomic alterations were color coded and the length of the bar represents the frequency of mutations (**A**). In total of 1534 ICCs from ten studies, 38 FGFR2 genetic alterations excluding fusions were found and the types of genomic alterations were color coded (**B**). A consortium of 91,129 multiple tumors were collected to identify FGFR2 in-frame deletions. In total, eleven tumor types that carried FGFR2 genetic short in frame deletions were identified with ICC at the highest frequency (0.62%, 7/1122), notched rectangles represent the frame deletion represented twice, the percentages represent the frequency of the mutation (**C**). Pan-cancer study of 9999 cases from public database was analyzed to evaluate the prevalence of FGFR2 site mutations, there were 70 site mutations in 65 patients were found, and in ICC the prevalence was 1.08% (**D**). Other tumors that may show scattered FGFR2 point mutations but are not listed in the figure include: cancer of unknown (1/120, A511T), uterine corpus endometrial carcinoma (1/61, S252W), extrahepatic cholangiocarcinoma (1/351, R255W), gallbladder carcinoma (1/240, N441S), gastric cancer (1/866, K399Q), ovarian cancer(1/261, S252W) and urothelial carcinoma (1/96,Q259L)

In addition to FGFR2 translocations, NSG also revealed four patients with intragenic mutations in 290 cases. Among these, one harbored P253R missense mutation and a frameshift deletion I548Wfs*8. R203C and C382R mutations in FGFR2 were respectively harbored by two different patients (Fig. 2A), which have been reported in ICC and other tumor types likely oncogenic [15, 19–21]. Besides, one patient harbored an in-frame deletion in the kinase domain (N631_M640del) of FGFR2 (Additional file 7: Table S2). Activating extracellular domain alterations have been identified in other oncogenic receptor tyrosine kinases, such as EGFR, HER2, PDGFRA, and RET [22–25]. Activated site mutations and extracellular domain in-frame deletions of FGFR2 were also identified in ICC [15] and craniosynostosis syndromes as well [26–29]. However, the in-frame deletion in the FGFR2 kinase domain was not reported previously.

We further analyzed a total of 1534 ICC patients in 10 studies from public databases for the distribution of FGFR2 mutation types in ICC. This led to the identification of 38 FGFR2 genetic alterations excluding fusions in 36 patients (2.33%) (Figs. 1, 2B, Additional file 8: Data S1). It is worth noting that, in addition to site mutations, 10 cases with FGFR2 deletion were identified, including short in-frame deletions (H167_N173del, S282_N297del, V280_K292del, and P286_K292del), single amino acid deletions (V233del and H624del), truncated mutations (Y561*) and frameshift mutations (V463 Gfs*3). Although most of the in-frame deletions happen in exon5 and exon7 that cause truncated extracellular domain, there are also in-frame deletions in exon 13 (H624del and Y561*) that cause truncated kinase domain in FGFR2 in ICC. These findings suggested that kinase domain deletion was not a random event in the ICC cohort.

Next, we analyzed the FGFR2in-frame deletions using data from a consortium of multiple tumors. This consortium collected tumor cases from across China and detected a total of 91,129 tumor patients for tumor mutations. In total, thirty-one cases from differenttumor types including ICC were identified to carry FGFR2 genetic short deletions in the consortium (Additional file 9: Data S2, Fig. 2C). FGFR2 in-frame deletion occurred in patients with ICC at the highest frequency (0.62%, 7/1122) compared with others, followed by lung squamous carcinoma (0.53%, 5/945) and endometrioid carcinoma (0.53%, 1/188). Those in-frame deletions are widely distributed, ranging from exon 4 to exon 17, in different tumor types. In ICC, the deletions were enriched in exon 7 with predominating mutations of I288-D304del and L258-D304del. Notably, there was also a deletion in the FGFR2 TK domain (p. K545del) in an ICC patient, which proved that the TK domain deletion was not a random event.

To further investigate whether the site mutations and truncated mutations of FGFR2 have cancer specificity, we studied a pan-cancer dataset of 9999 cases from a public database. In a total of 9999 cases, 70 FGFR2 site mutations were found in 65 patients (Fig. 2D, Additional file 10: Data S3), distributed over all exons without hotspot mutations. The highest frequency of mutations was observed in patients with uterine corpus endometrial carcinoma at (9.84%, 6/61), and the prevalence in ICC was 1.08%, which was similar to the results in our cohort (3/290, 1.03%).

Overall, these findings suggest that in comparison to other malignancies, the occurrence rate of FGFR2 in-frame deletion is the highest in ICCs (0.62%, 7/1122), with predominating mutations of I288-D304del and L258-D304del, and kinase domain in-frame deletions are not a random event.

### Clinicopathologic features of ICCs with FGFR2 genetic alterations

**Fig. 3:**
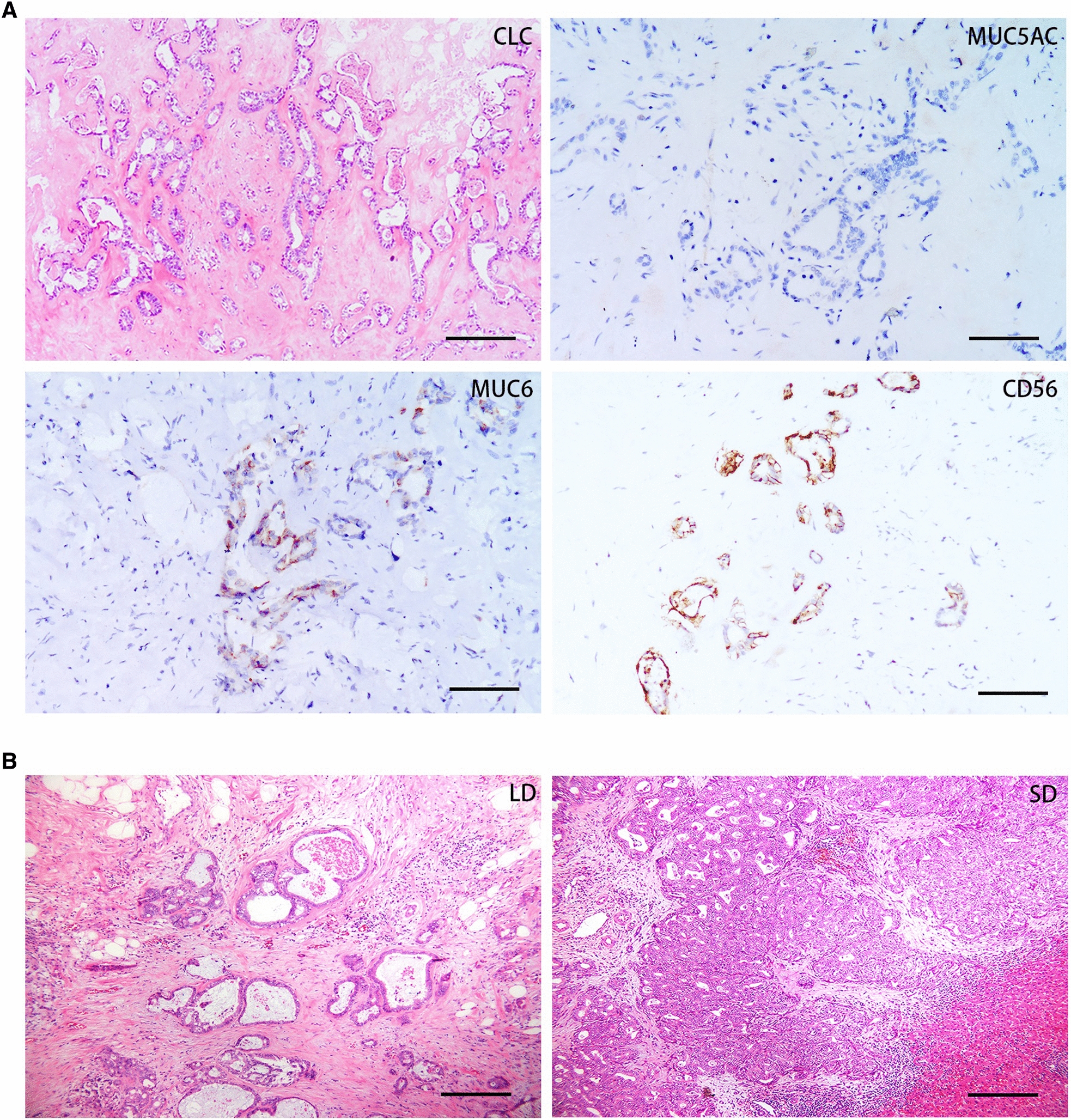
Histopathological features of FGFR2 genetic alteration ICCs. FGFR2 gene fusion/translocation ICCs were enriched for specific subtypes in small duct cholangiocarcinoma (CLC) with specific immunological features showing MUC5A negativity, MUC6 negativity or sporadic positivity, and CD56 positivity. HE and IHC staining are presented at 200×, the scale represents 100 µm (**A**). ICCs with FGFR2 site mutations, in-frame deletion and frame-shift deletion presented large duct (LD) or small duct (SD) in histological subtypes. HE staining are presented at 200×, the scale represents 100 µm (**B**)

The clinicopathologic and molecular features of ICC cases with FGFR2 fusion/translocation or non-fusion/translocation mutations are summarized in Additional file 7: Table S3. In addition to gross classification and histological classification, there was no significant difference in age, risk factors, tumor number and size, tumor differentiation or clinical stage between translocation-positive and translocation-negative cases. Morphologically, 29 of 30 tumors represented mass-forming (MF) growth in gross classification, and only one tumor represented intraductal growth (IG), which showed MF type predominance. Microscopically, twenty-nine cases were adenocarcinomas, and one was an adenosquamous carcinoma. Most notably, of the twenty-nine adenocarcinomas, FGFR2 gene fusions were specifically enriched in cholangiolocarcinoma (CLC), with 79.3% (23/29) of cases displaying CLC. All these FGFR2 fusion/translocation CLC cases also displayed similar histopathological and immune subtypes: tumor cells showed small cuboids with a high nuclear/cytoplasmic ratio, oval nuclei, pale cytoplasm, and small atypia and were mostly in a well-differentiated state, with loose formation in the hyalinized collagen fibrous stroma characterized by angular small ducts, cords, or branching arrangements, no or few mucus secretions, MUC5ac negativity, MUC6 negativity or sporadic positivity, and CD56 positivity (Fig. 3A). Given the unique gross, morphological and molecular features of FGFR2 fusion cases, we regarded FGFR2 fusion/translocation tumors as a unique class of ICC, which we refer to as the “FGFR2 fusion subtypes of ICC”.

Because of the limited sample sizes, we did not compare the clinicopathologic molecular features between the FGFR2 mutation-positive and FGFR2 mutation-negative groups. The clinicopathologic and molecular features of the four FGFR2 mutation cases are summarized in Additional file 7: Table S2. Different from FGFR2 fusion/translocation, only half of the tumors with FGFR2 mutation represented simple mass-forming (MF) growth in gross classification, and the other half represented gross classification with mixed MF and PI. Similarly, half of the tumors with FGFR2 mutation showed the small duct (SD) type, and the other half showed the large duct (LD) type (Fig. 3B).

### Co-mutations occur in FGFR2-mutated ICCs

**Fig. 4:**
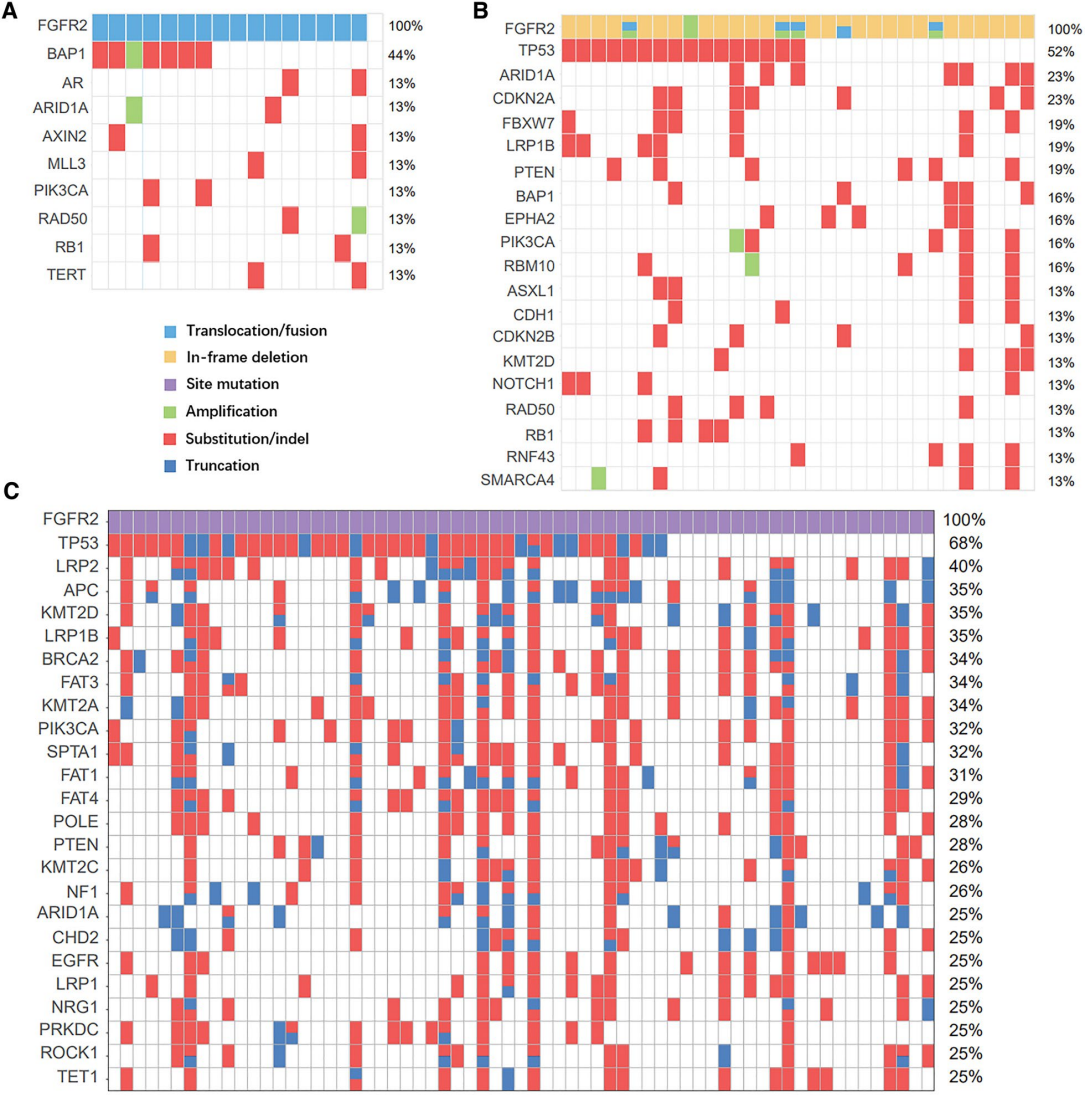
Underlying co-mutation features of different types of FGFR2 genetic alterations. Comutation plot of seventeen FGFR2 translocation/fusion cases (**A**), thirty-one FGFR2 in-frame deletion cases (**B**) and sixty-five FGFR2 site mutation cases (**C**)

Detailed information on genetic alternations in 17 FGFR2 translocation/fusion cases, 31 FGFR2 in-frame deletion cases, and 65 FGFR2 site mutation cases are summarized in Additional file 11: Data S4, Additional file 12: Data S5, and Additional file 13: Data S6. The visualized genetic data for these cases are summarized in Fig. 4. In FGFR2 translocation/fusion cases, there was no co-occurring TP53 driver mutation. However, TP53/CDKN2A driver mutations were enriched in the FGFR2 in-frame deletion and site mutation cases. Combined with the FISH results (Additional file 1: Fig. S1) and the distinct histological morphology exhibited by cases with FGFR2 translocations/fusions, it can be inferred that the reliance of FGFR2 translocation/fusion on potential oncogenic factors, such as TP53 mutations, is minimal. On the other hand, the oncogenic potential of FGFR2 in-frame deletions and point mutations seems to be less pronounced as compared to that of FGFR2 fusions, indicating a difference in their carcinogenic capacity. This is significantly underscored by the observation that 52% of FGFR2 in-frame and 68% of FGFR2 site mutations co-occur with TP53 mutations.

### Various oncogenic activities and diverse responses to FGFR-selective small molecule kinase inhibitor (SMKI) among FGFR2 mutants

**Fig. 5:**
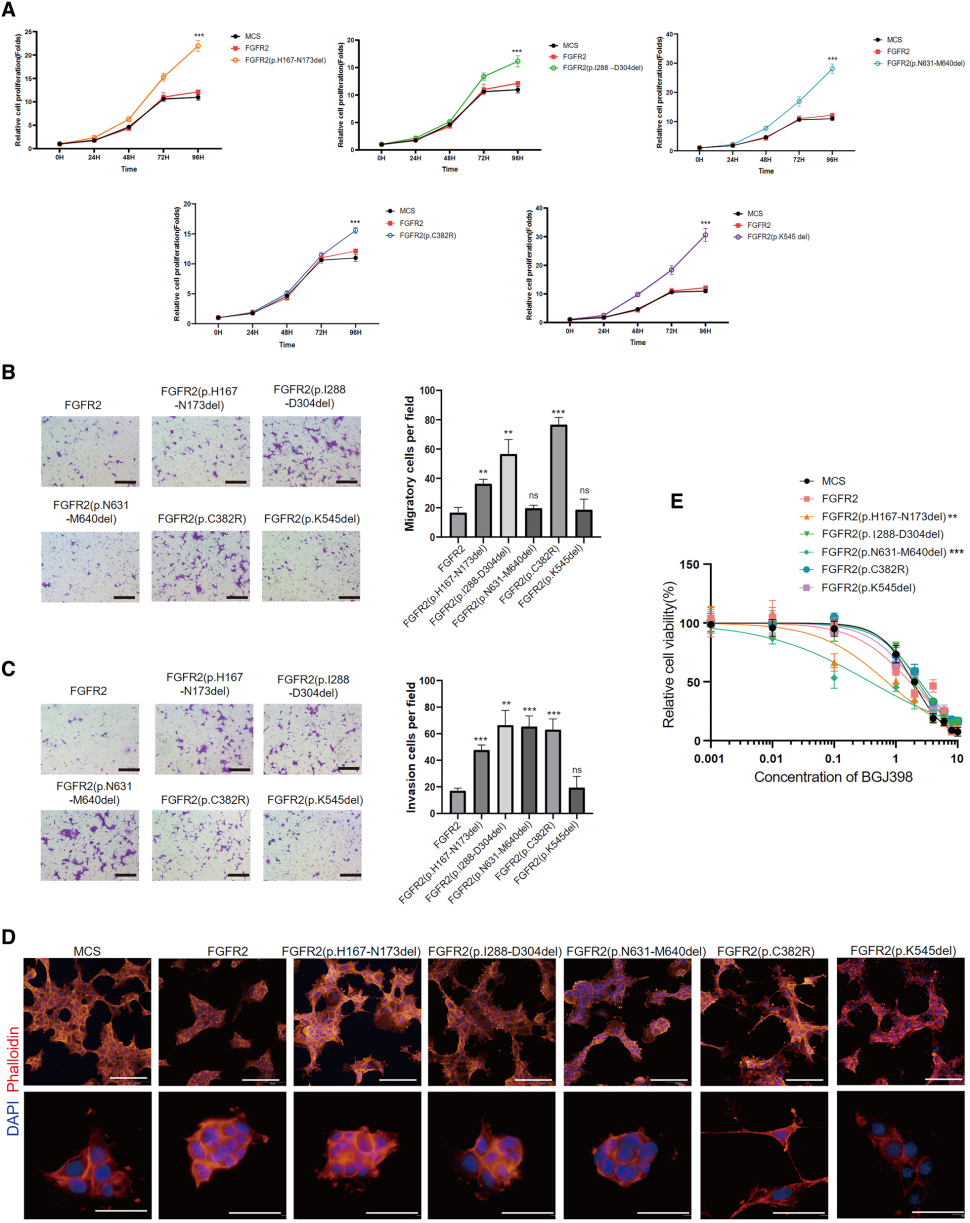
Various oncogenic activities and diverse responses to FGFR-selective small molecule kinase inhibitor (SMKI) among FGFR2 mutants. Proliferation activities of AML12 cells expressing MCS, FGFR2 and different FGFR2 mutants are shown (**A**). Representative images of transwell migration assay and average numbers of migrated AML12 cells expressing FGFR2 and different FGFR2 mutants are shown (**B**), the scale represents 100 µm. Representative images of invasion assay and average number of invasive AML12 cells expressing FGFR2 and various FGFR2 mutants are shown (**C**). Cellular skeleton staining revealed the morphological changes in AML12 cells when expressing Lenti-CMV-MCS control virus (MCS), FGFR2 and different FGFR2 mutants (**D**). Evaluation the sensitivity of RBE cells with different FGFR2 mutants to BGJ398 (**E**)

Since FGFR2 fusion has been demonstrated to be an independent driving factor for the occurrence of intrahepatic cholangiocarcinomas (ICCs), the impact of other mutations on ICC remains unknown. To assess the function of various non-fusion FGFR2 mutants and determine their sensitivity to FGFR2-targeted drugs, we selected representative in-frame deletion and site mutation mutants (FGFR2 p.H167-N173del, p.I288-D304del, p.N631-M640del, p.C382R, and p.K545del), prepared lentiviruses expressing these mutants which labeled with FLAG epitope tag (Additional file 2: Fig. S2). We introduced these mutants into AML12 cells, the normal mouse hepatical cell lines, to assay their ability in cell proliferation, migration, and invasion. These non-fusion mutants promoted the proliferation of AML12 cells according to CCK-8 assay (Fig. 5A). Transwell assays were performed to determine the migratory and invasive capacities of AML12 cells. Nearly all mutants enhanced the migratory and invasive abilities of AML12 cells (Fig. 5B, C). The capacities of these mutants in cell proliferation, migration, and invasion were also tested in human intrahepatic biliary epithelial cells HIBEC and mouse fibroblast cells NIH3T3 (Additional file 3: Fig. S3A–C, Additional file 4: Fig. S4A–C). It is noticed that the introduction of p.C382R mutant changed the cell morphology. Phalloidin cytoskeleton staining revealed that cells expressing this mutant exhibited notably bundled cellular skeleton structures (Fig. 5D, Additional file 4: Fig. S4D), suggesting that FGFR2 mutations may increase cellular motility. In our pursuit to determine whether FGFR2 mutations could increase the therapeutic sensitivity of ICC cells, we employed human cholangiocarcinoma RBE cells as a model system and infected them with lentiviruses expressing a range of FGFR2 mutations. FGFR inhibitors can be broadly categorized into FGFR-selective small molecule kinase inhibitors (SMKIs) and non-selective SMKIs. The former group includes BGJ398, ARQ 087, AZD4547 [30], and CH5183284/Debio 1347, while the latter group encompasses Ponatinib, Pazopanib and Trametinib [31]. The therapeutic targeting of FGFRs with SMKIs has emerged as a promising personalized treatment strategy specifically for patients with FGFR2 mutations. Among these inhibitors, several studies have highlighted that FGFR genetic aberrations exhibit high sensitivity to BGJ398 [32, 33]. Furthermore, Infigratinib (BGJ398) has demonstrated encouraging outcomes in a multicenter, open-label, single-arm, phase 2 trial, establishing its therapeutic efficacy for previously treated patients with advanced or metastatic cholangiocarcinoma harboring FGFR2 fusions or rearrangements [34]. The US FDA has also granted approval to pemigatinib for the management of advanced cholangiocarcinoma patients characterized by fibroblast growth factor receptor 2 (FGFR2) translocations [4]. So in our study, we select BGJ398 to evaluate the sensitivity of FGFR2 mutants. Our observations revealed that exclusively the cells containing FGFR2 p.H167-N173del and p.N631-M640del mutations showed considerable sensitivity to BGJ398 (Fig. 5E). Therefore, although FGFR2 mutants exhibit oncogenic activity, not all mutants are sensitive to targeted therapies. It is crucial to determine the sensitivity of different mutant types prior to clinical treatment with targeted therapies.

### Frameshift mutation resulting in premature stop codons is also a potential therapeutic target

**Fig. 6:**
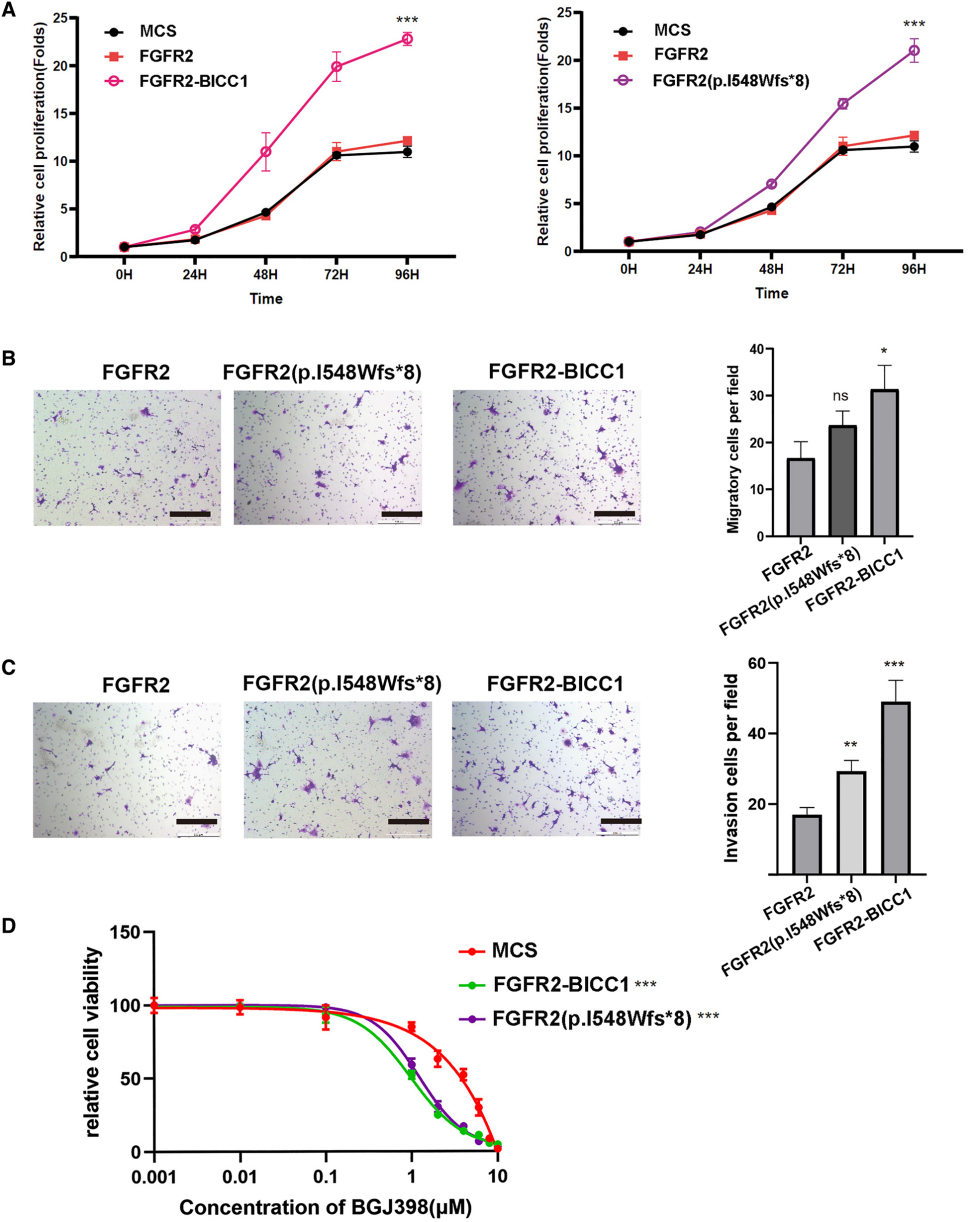
Frameshift mutation resulting in premature stop codons is also a potential therapeutic target. Proliferation activities of AML12 cells expression Lenti-CMV-MCS control virus (MCS), FGFR2, FGFR2-BICC1 fusion and FGFR2 I548Wfs*8 mutant are shown (**A**). Representative images of transwell migration and average numbers of migrated cells expressing above three clones (**B**), the scale represents 100 µm. Representative images of invasion and average colonies of invasion cells expressing above three clones in AML12 cells are shown (**C**). Capability of FGFR2 I548Wfs*8 rendering RBE cells sensitive to BGJ398 (**D**)

Both our study cohort and multi-center research cohorts have identified the presence of FGFR2 frameshift mutations. These mutations often lead to premature termination codons, resulting in an incomplete FGFR2 TK domain. However, the oncogenic activity and sensitivity of these frameshift mutations to targeted therapies remain uncertain. In this study, we focused on the I548Wfs*8 mutation, which we discovered, and developed lentiviruses expressing the mutant. We used FGFR2-BICC1 fusion as a positive control and empty vector as a negative control in our experiment, we introduced these lentiviruses into AML12, HIBEC and NIH3T3 cells to evaluate the ability of I548Wfs*8 to induce cell proliferation, invasion, migration (Fig. 6A–C, Additional file 5: Fig. S5A–C, Additional file 6: Fig. S6A–C), and sensitivity to targeted drugs (Fig. 6D). Our findings demonstrate that FGFR2 frameshift mutant I548Wfs*8 exhibits oncogenic activity and sensitivity to FGFR inhibitors.

## Discussion

The occurrence and mortality rates of cholangiocarcinoma have risen in recent years, particularly for intrahepatic cholangiocarcinoma (ICC) [35–37]. Traditionally, surgical intervention was the solitary effective treatment strategy for ICC, but this was often an unattainable option for advanced-stage cases. Presently, in the rapidly-evolving landscape of targeted cancer therapeutics, several drugs have garnered regulatory approval for the treatment of ICC. Among these, Pemigatinib (BGJ398) has been approved for use in ICC patients presenting with FGFR2 fusion, while Tibsovo has received approval for treating ICC patients carrying an IDH1 mutation [38]. As FGFR2 fusion/translocation has been identified as a common event and is present at much higher frequencies in ICC [6–12], studies have emphasized on FGFR2. In our study, through FISH and/or NGS, we identified 30 cases harboring FGFR2 fusion/translocation. Clinicopathologically, patients harboring FGFR2 fusion/translocation did not show age/gender or tumor grade and tumor differentiation preponderance. Nevertheless, in other studies, researchers found that ICC patients with FGFR2 gene fusions were younger with a female preponderance [39]. Notably, we found that cases with FGFR2 gene fusion/translocation tended to have MF in gross classification and small ducts in histological classification, suggesting that the presence of FGFR2 gene fusion/translocation may have prognostic utility. Based on mucin productivity and immunophenotype, ICC was classified into large ducts and small ducts. Small duct-type ICC is generally characterized by little mucin production and exhibits immunoreactivity to N-cadherin and/or NCAM. More importantly, IDH mutation and FGFR2 translocation are restricted to small duct-type ICC, and small duct-type ICC often has prognostic utility [40, 41]. Most importantly, we observed that FGFR2 fusion/translocation tended to be enriched in special types of small duct ICC- cholangiolocarcinoma (CLC). These cases represent similar histological features: tumor cells show a small cuboid with a high nuclear/cytoplasmic ratio, oval nucleus, pale cytoplasm, lack of mucus, small atypia, and mostly in a well-differentiated state, with loose formation in the hyalinized collagen fibrous stroma characterized by angular small ducts, cords, or branching arrangements. Immunohistochemistry displayed MUC5AC negativity, MUC6 negativity or sporadic positivity, and CD56 was often positive. CLC arises in small intrahepatic ductules and only accounts for ~ 10% of ICC [42, 43], characterized by low-grade cytologic atypia, anastomosing cords and glands resembling cholangioles or canals of Hering [44]. In our study, 79.3% (23/29) of FGFR2 fusion/translocation ICCs displayed CLC with a similar immunotype. Because of the unique gross and morphological features of FGFR2 fusion cases, we described this distinct group as “FGFR2 fusion subtype ICC”.

Our genomic investigation of ICC underscores the remarkable significance of FGFR2 mutations, including site mutations and short in-frame deletions. We observed five types of mutations across a total of four patients, encompassing three site mutations (p. R203C, p. P253R, and p. C382R), one in-frame deletion (p. N631_M640del), and one frame-shift (p.I548Wfs*8), all located within the Tyrosine Kinase (TK) domain of FGFR2. In contrast to FGFR2 fusion/translocation cases, half of the tumors with FGFR2 mutations exhibited a mixed gross classification of MF and PI, and all of these mixed cases displayed a large duct (LD) type in histological classification. Furthermore, FGFR2 mutations demonstrated a prevalence in advanced stages, as opposed to a dominance in earlier stages observed in FGFR2 fusion/translocation cases. Notably, FGFR2 site mutations within the extracellular domain are acknowledged as oncogenic mutations driving FGFR signaling activation [21]. For instance, FGFR2 F276C has been reported as a target site in ICC [15, 16, 20]. Although all the site mutations we identified were situated in the FGFR2 extracellular region, pan-cancer study outcomes reveal that FGFR2 mutation sites are distributed across all exons. Given that activating FGFR2 mutations are exceptionally rare in ICC, future tumor-agnostic investigations focusing on FGFR site mutations harbor the potential to expand the applicability of FGFR inhibitors. It is worth mentioning that we also identified two TK domain FGFR2 deletions that have never been reported in other studies; one harbored an EX14 in-frame deletion (FGFR2 p. N631_M640del), while the other harbored an EX12 out-frame deletion (FGFR2 p. I548 Wfs*8), which results in premature termination of the protein at exon 12 during translation. FGFR2 extracellular domain in-frame deletions are known to cause autosomal dominant congenital craniosynostosis syndromes during growth [26–29] and cause oncogenicity during cancer development in ICC [15], whereas the function of FGFR2 TK domain deletions was not known until now.

Data derived from a consortium of multi-central studies across various tumors identified deletions within the FGFR2 Tyrosine Kinase (TK) domain. Similarly, our findings also marked the presence of FGFR2 deletions across multiple tumor types. Interestingly, short in-frame deletions in FGFR2 genetic structure were observed at a higher frequency in ICC patients as compared to other cancer types, with lung squamous carcinoma following closely. The distribution of FGFR2 deletion mutations spanned a wide range, from exon 4 through to exon 17. Both in ICC and lung squamous carcinoma, the deletion mutation was predominantly observed in FGFR2 EX7. However, in ICC, the mutations were primarily concentrated in the L258-D304 del area, while in lung squamous carcinoma, the mutation hotspot was R251-V274 del. Intriguingly, in the ICC cohort, deletions were also noted in the FGFR2 TK domain, substantiating the non-random nature of the TK domain deletion occurrence within our cohort. Data from the multi-center consortium represented in ICC revealed that p. K545del was located within the FGFR2 TK domain. Site mutations within this FGFR2 TK domain have been implicated in molecular brake mechanism and have been observed to instigate clinical resistance against FGFR inhibitors. These site mutations include p. V564, p. E565, p. N594, p. L617, p. K641, and p. L659 [20, 45]. p. K545del and p. N631-M640del were very close to these polyclonal resistance mutation sites, and whether the deletion in the TK domain functioned as drug-resistant or drug-sensitive to FGFR inhibitors is unknown.

To assess the function of different FGFR2 mutants and determine their responsiveness to FGFR2-targeted drugs, we selected representative mutants (FGFR2 p.H167-N173del, p.I288-D304del, p.N631-M640del, p.C382R, p.K545del and p. I548 Wfs*8), constructed expression vectors of these mutants and introduced these mutants into AML12, HIBEC and NIH3T3 cells for assaying their oncogenic abilities. We found although nearly all FGFR2 mutants we selected have the propensity to promote tumorigenesis and metastasis of ICC, not all of the mutants are capable of rendering ICC cells sensitive to targeted therapy. It has been found that although nearly all activating FGFR2 mutants can transform and activate the receptor, the mechanisms of kinase activation are completely different. In the case of FGFR2 gene fusion, the tyrosine kinase domain of FGFR2 is retained but the C-terminal tail is replaced by a partner gene, which usually contains an intracellular dimerization domain that promotes FGFR2 dimerization and kinase activation [13]. In the case of FGFR2 EIDs, the intracellular domain of FGFR2 is intact, but changes in the extracellular domain of FGFR2 cause cysteine residues to be lost or gained. The changes in cysteine residues disrupt inhibitory intramolecular disulfide bonds or form abnormal intermolecular disulfide bonds, which promote FGFR2 dimerization and kinase activation [46]. Truncation of FGFR2 in the post kinase region is sufficient to drive ligand-independent growth and represent an alternative mechanism of FGFR2 activation, it is distinct from fusions [47–49].

In summary, our investigation delved into FGFR2 genetic alterations in ICC and other malignancies. We discovered that the majority of FGFR2 fusions/translocations in ICC were predominantly observed in a subtype known as cholangiolocarcinoma, which we refer to as “FGFR2 fusion subtype ICC”. Among ICC patients, only 1.38% (4/290) exhibited FGFR2 mutations, with FGFR2 in-frame deletions being more prevalent in ICC compared to other cancer types. While FGFR2 fusions were found to infrequently coincide with other driver genes, FGFR2 in-frame deletions and point mutations were frequently observed in conjunction with TP53 mutations in ICC. The various FGFR2 mutations exhibited diverse responses to FGFR-selective small molecule kinase inhibitors in ICC cells, thereby highlighting the need to consider unique mutation profiles when devising targeted therapeutics. Our findings provide valuable insights for the development of future interventions tailored to patients with FGFR2 genetic aberrations in ICC.

### Supplementary Information

**Additional file 1: Figure S1.** Schematic representation of FGFR2 gene translocation. Green and red spots indicate the genomic location of 5′ and 3′ FISH probes for the FGFR2 gene. Distinct orange and green signals (**A**) or signal orange (**B**) in more than 20% of the tumor cells represent FGFR2 rearrangement.

**Additional file 2: Figure S2.** Expression of different FGFR2 mutants. The cDNAs of different FGFR2 mutants were transfected into NIH3T3 cells. The expression of mutants was detected by Western blot with anti-Flag antibody.

**Additional file 3: Figure S3.** Proliferation, migration and invasion activities of different FGFR2 mutants in HIBEC cells. Proliferation activities of HIBEC cells expressing Lenti-CMV-MCS control virus (MCS) and different FGFR2 mutants are shown (**A**). Representative images of transwell migration assay and average numbers of migrated HIBEC cells expressing MCS and different FGFR2 mutants are shown (**B**), the scale represents 100 µm. Representative images of invasion assay and average number of invasive HIBEC cells expressing MCS and various FGFR2 mutants are shown (**C**).

**Additional file 4: Figure S4.** Proliferation, migration and invasion activities of different FGFR2 mutants in NIH3T3 cells. Proliferation activities of NIH3T3 cells expression Lenti-CMV-MCS control virus (MCS), FGFR2 and different FGFR2 mutants are shown (**A**). Representative images of transwell migration and average numbers of migrated NIH3T3 cells expressing FGFR2 and different FGFR2 mutants are shown (**B**), the scale represents 100 µm. Representative images of invasion assay and average colonies of invasion NIH3T3 cells expressing FGFR2 and different FGFR2 mutants are shown (**C**). Cellular skeleton staining revealed the morphological changes in NIH3T3 cells when expressing MCS, FGFR2 and different FGFR2 mutants (**D**).

**Additional file 5: Figure S5.** Proliferation, migration and invasion activities of FGFR2(p. I548 Wfs*8) in HIBEC cells. Proliferation activities of HIBEC cells expression Lenti-CMV-MCS control virus (MCS), FGFR2-BICC1 fusion and FGFR2(p. I548 Wfs*8) shown (**A**). Representative images of transwell migration and average numbers of migrated HIBEC cells expressing Lenti-CMV-MCS control virus (MCS), FGFR2-BICC1 fusion and FGFR2( p. I548 Wfs*8) are shown (**B**), the scale represents 100 µm. Representative images of invasion assay and average colonies of invasion HIBEC cells expressing MCS, FGFR2-BICC1 fusion and FGFR2(p. I548 Wfs*8) are shown (**C**).

**Additional file 6: Figure S6.** Proliferation, migration and invasion activities of FGFR2(p. I548 Wfs*8) in NIH3T3 cells. Proliferation activities of NIH3T3 cells expression Lenti-CMV-MCS control virus (MCS), FGFR2-BICC1 fusion and FGFR2(p. I548 Wfs*8) shown (**A**). Representative images of transwell migration and average numbers of migrated NIH3T3 cells expressing MCS, FGFR2-BICC1 fusion and FGFR2(p. I548 Wfs*8) are shown (**B**), the scale represents 100 µm. Representative images of invasion assay and average colonies of invasion NIH3T3cells expressing MCS, FGFR2-BICC1 fusion and FGFR2(p. I548 Wfs*8) are shown (**C**).

**Additional file 7: Table S1.** Clinicopathologic, molecular Features and Follow-up Information in Patients with FGFR2 fusion. **Table S2.** Clinicopathologic, molecular Features and Follow-up Information in Patients with FGFR2 mutation. **Table S3.** Clinicopathologic Features between FGFR2 fusion/translocation positive and negative cases.

**Additional file 8:** 38 FGFR2 genetic alterations excluding fusions in 36 ICC patients from public databases.

**Additional file 9:** 65 FGFR2 in-frame deletions cases searched in total 91,129 cases.

**Additional file 10:** 31 FGFR2 site mutations searched in total 9999 cases from public database.

**Additional file 11:** Detailed information on genetic alternations in 17 FGFR2 translocation/fusion cases.

**Additional file 12:** Detailed information on genetic alternations in 65 FGFR2 site mutation cases.

**Additional file 13:** Detailed information on genetic alternations in 31 FGFR2 in-frame deletion cases.

## Data Availability

The datasets used or analyzed during the current study are available from the corresponding author on reasonable request.
