# Supplementary figures and images for: Oncogenic activation revealed by FGFR2 genetic alterations in intrahepatic cholangiocarcinomas
Source: Cell Biosci. 2023 Nov 14;13:208. doi: 10.1186/s13578-023-01156-7 (PMC10644541; doi:10.1186/s13578-023-01156-7)

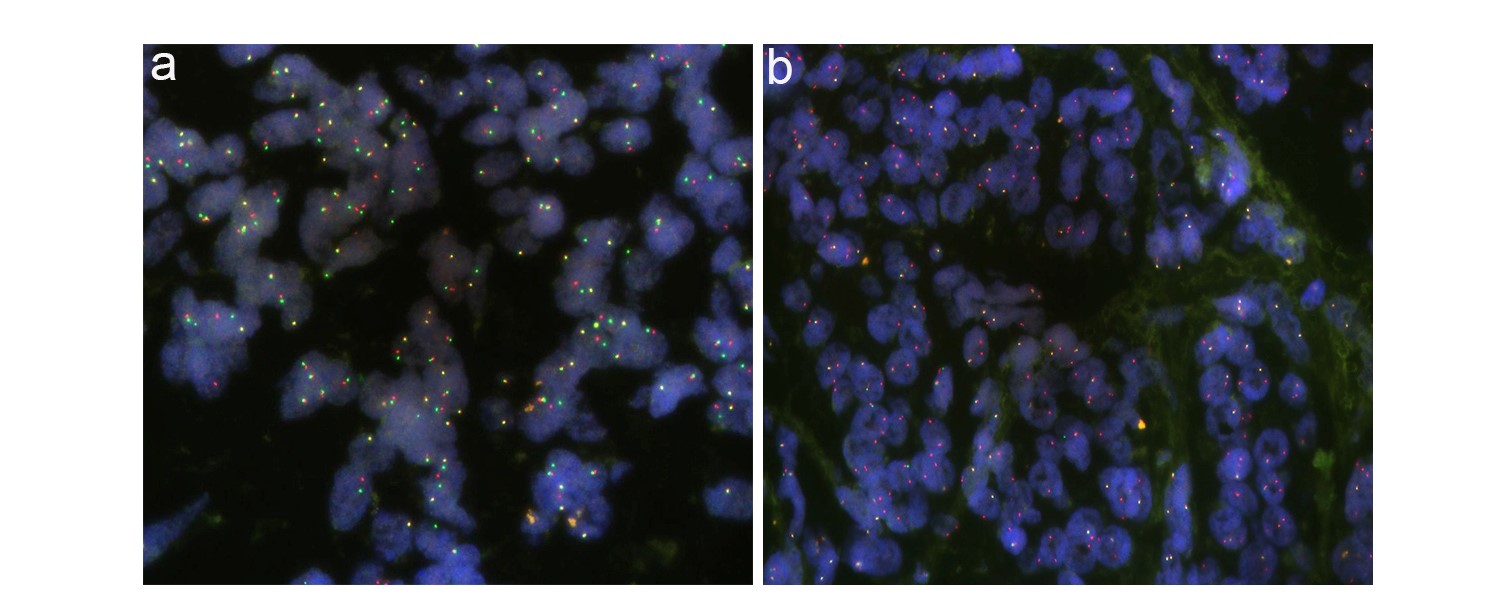

Supplement: Supplementary file 1 — Additional file 1: Figure S1. Schematic representation of FGFR2 gene translocation. Green and red spots indicate the genomic location of 5′ and 3′ FISH probes for the FGFR2 gene. Distinct orange and green signals (A) or signal orange (B) in more than 20% of the tumor cells represent FGFR2 rearrangement. [file 13578_2023_1156_MOESM1_ESM.jpg]

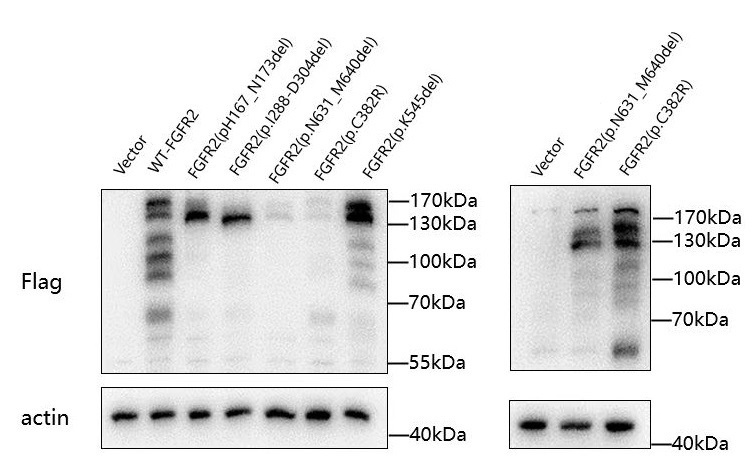

Supplement: Supplementary file 2 — Additional file 2: Figure S2. Expression of different FGFR2 mutants. The cDNAs of different FGFR2 mutants were transfected into NIH3T3 cells. The expression of mutants was detected by Western blot with anti-Flag antibody. [file 13578_2023_1156_MOESM2_ESM.jpg]

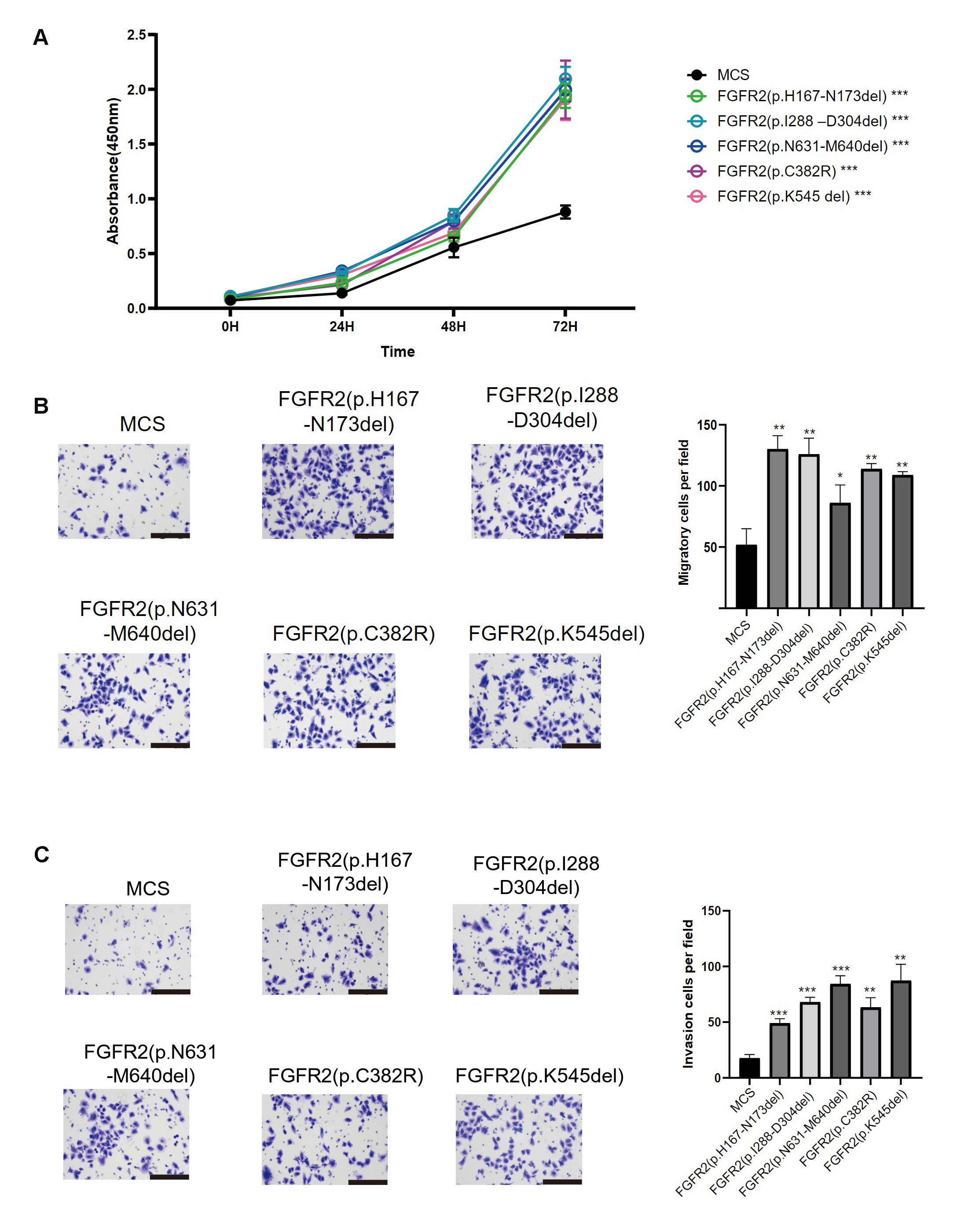

Supplement: Supplementary file 3 — Additional file 3: Figure S3. Proliferation, migration and invasion activities of different FGFR2 mutants in HIBEC cells. Proliferation activities of HIBEC cells expressing Lenti-CMV-MCS control virus (MCS) and different FGFR2 mutants are shown (A). Representative images of transwell migration assay and average numbers of migrated HIBEC cells expressing MCS and different FGFR2 mutants are shown (B), the scale represents 100 µm. Representative images of invasion assay and average number of invasive HIBEC cells expressing MCS and various FGFR2 mutants are shown (C). [file 13578_2023_1156_MOESM3_ESM.jpg]

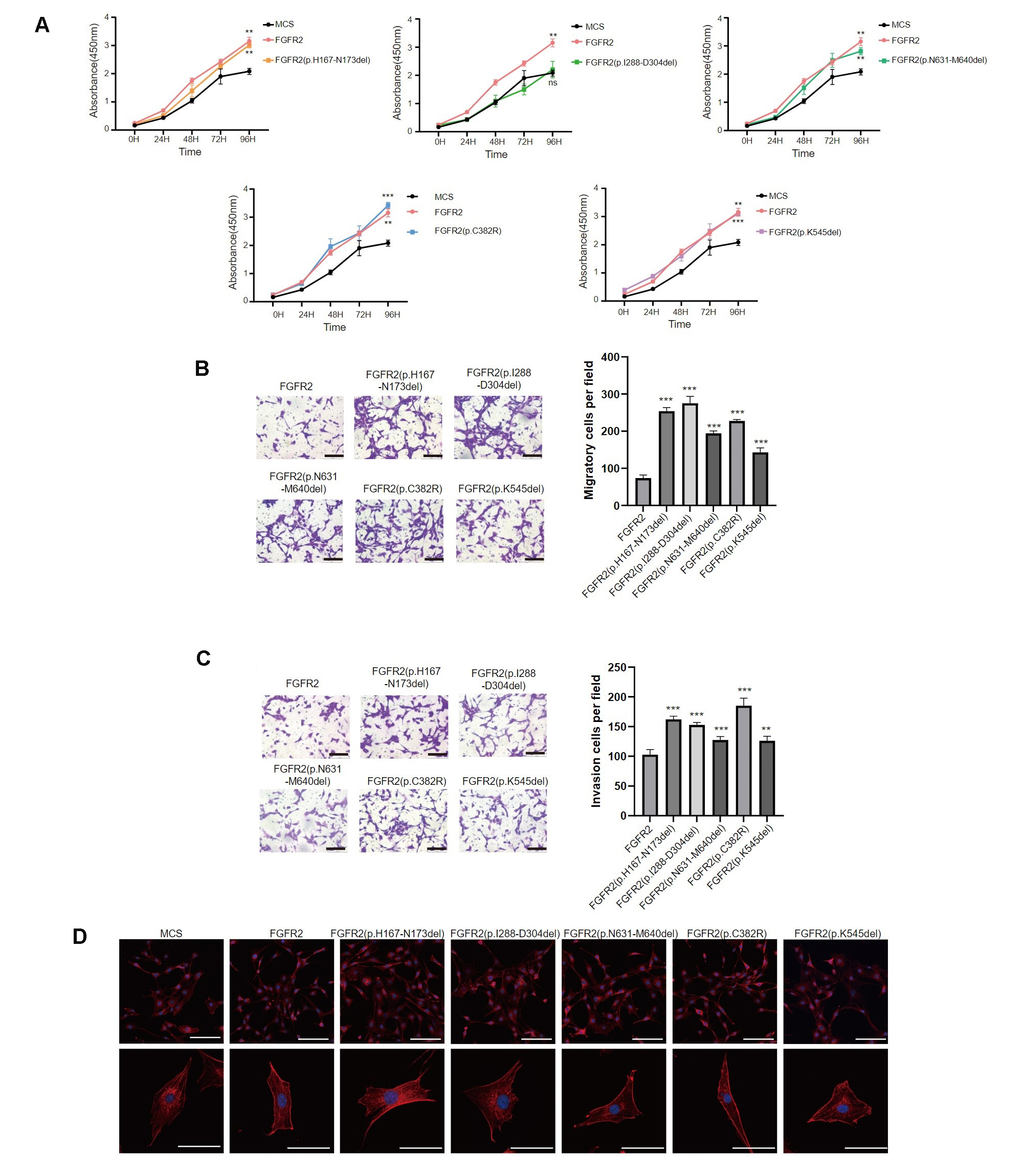

Supplement: Supplementary file 4 — Additional file 4: Figure S4. Proliferation, migration and invasion activities of different FGFR2 mutants in NIH3T3 cells. Proliferation activities of NIH3T3 cells expression Lenti-CMV-MCS control virus (MCS), FGFR2 and different FGFR2 mutants are shown (A). Representative images of transwell migration and average numbers of migrated NIH3T3 cells expressing FGFR2 and different FGFR2 mutants are shown (B), the scale represents 100 µm. Representative images of invasion assay and average colonies of invasion NIH3T3 cells expressing FGFR2 and different FGFR2 mutants are shown (C). Cellular skeleton staining revealed the morphological changes in NIH3T3 cells when expressing MCS, FGFR2 and different FGFR2 mutants (D). [file 13578_2023_1156_MOESM4_ESM.jpg]

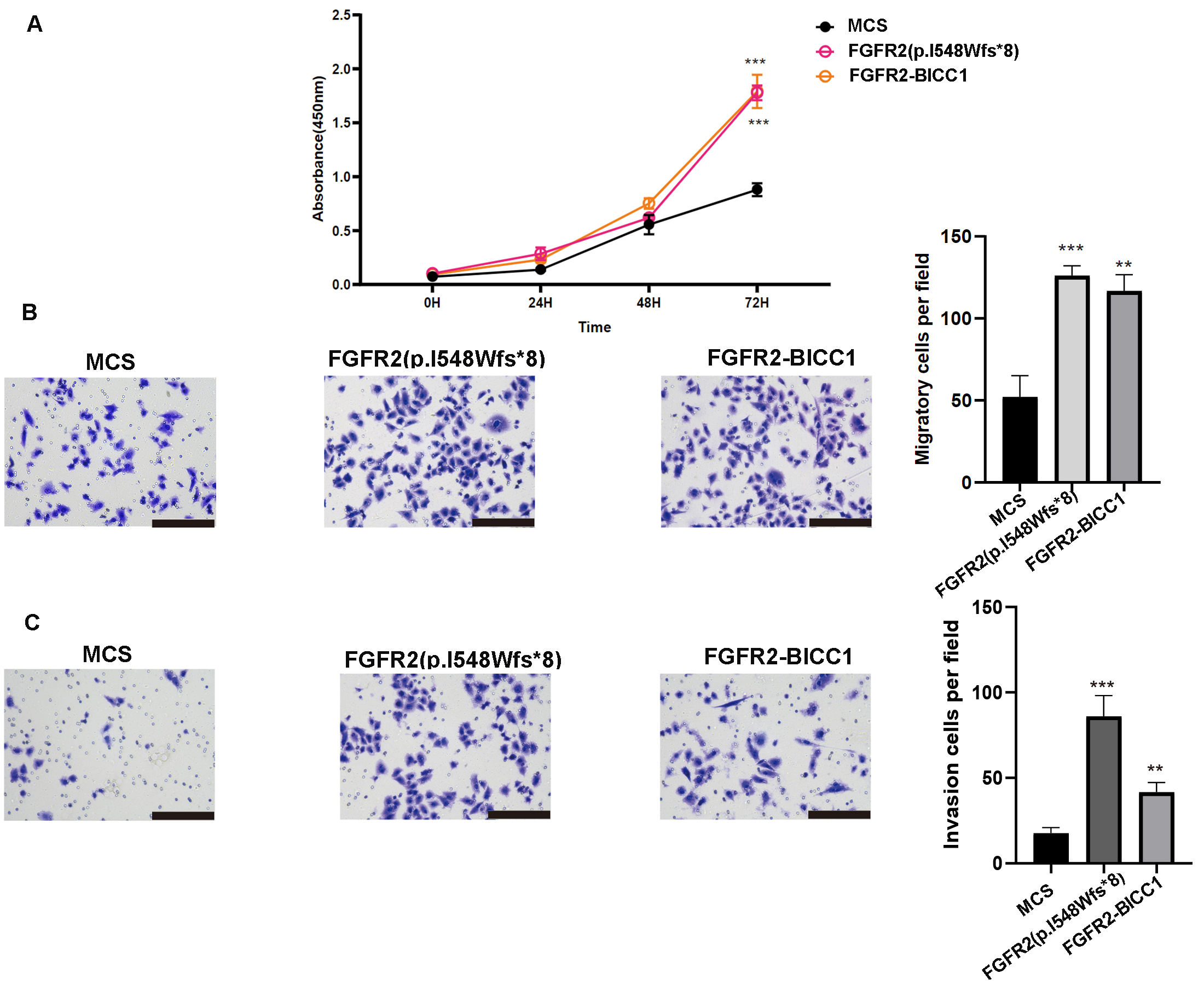

Supplement: Supplementary file 5 — Additional file 5: Figure S5. Proliferation, migration and invasion activities of FGFR2(p. I548 Wfs*8) in HIBEC cells. Proliferation activities of HIBEC cells expression Lenti-CMV-MCS control virus (MCS), FGFR2-BICC1 fusion and FGFR2(p. I548 Wfs*8) shown (A). Representative images of transwell migration and average numbers of migrated HIBEC cells expressing Lenti-CMV-MCS control virus (MCS), FGFR2-BICC1 fusion and FGFR2( p. I548 Wfs*8) are shown (B), the scale represents 100 µm. Representative images of invasion assay and average colonies of invasion HIBEC cells expressing MCS, FGFR2-BICC1 fusion and FGFR2(p. I548 Wfs*8) are shown (C). [file 13578_2023_1156_MOESM5_ESM.jpg]

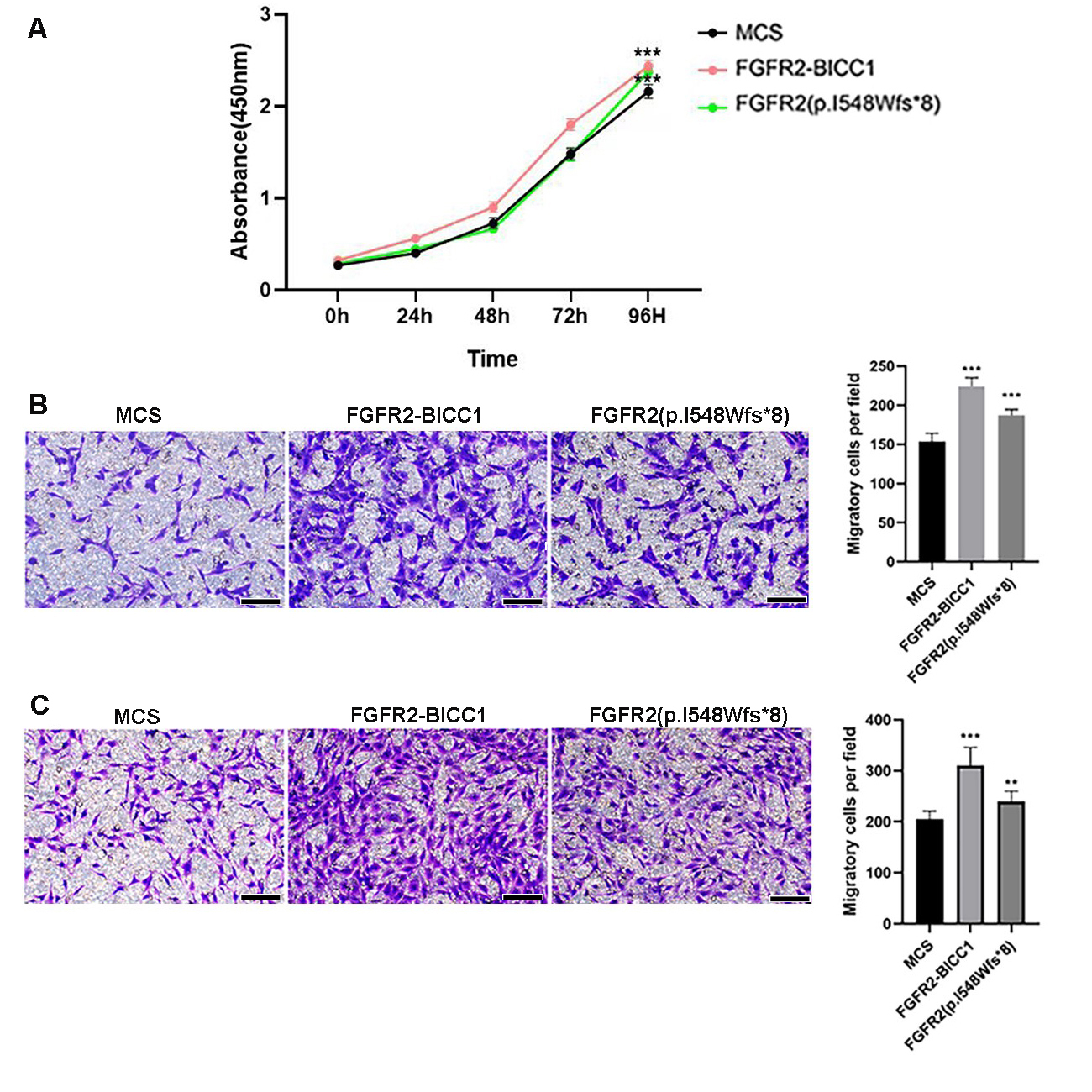

Supplement: Supplementary file 6 — Additional file 6: Figure S6. Proliferation, migration and invasion activities of FGFR2(p. I548 Wfs*8) in NIH3T3 cells. Proliferation activities of NIH3T3 cells expression Lenti-CMV-MCS control virus (MCS), FGFR2-BICC1 fusion and FGFR2(p. I548 Wfs*8) shown (A). Representative images of transwell migration and average numbers of migrated NIH3T3 cells expressing MCS, FGFR2-BICC1 fusion and FGFR2(p. I548 Wfs*8) are shown (B), the scale represents 100 µm. Representative images of invasion assay and average colonies of invasion NIH3T3cells expressing MCS, FGFR2-BICC1 fusion and FGFR2(p. I548 Wfs*8) are shown (C). [file 13578_2023_1156_MOESM6_ESM.jpg]
