# Supplementary material for: Oncogenic activation revealed by FGFR2 genetic alterations in intrahepatic cholangiocarcinomas
Source: Cell Biosci. 2023 Nov 14;13:208. doi: 10.1186/s13578-023-01156-7 (PMC10644541; doi:10.1186/s13578-023-01156-7)
Supplement: Supplementary file 7 — Additional file 7: Table S1. Clinicopathologic, molecular Features and Follow-up Information in Patients with FGFR2 fusion. Table S2. Clinicopathologic, molecular Features and Follow-up Information in Patients with FGFR2 mutation. Table S3. Clinicopathologic Features between FGFR2 fusion/translocation positive and negative cases. [file 13578_2023_1156_MOESM7_ESM.doc]

**Table S1** Clinicopathologic, molecular Features and Follow-up Information in Patients with FGFR2 fusion

| **Case** | **Age/**  **Gender** | **Fusion mode** | **Tumor size**  **(cm)** | **Gross classification** | **Histological classification** | **MUC**  **5AC** | **MUC6** | **CD56** | **Tumor Grade** | **Tumor differentiation** | **Follow-up** | **Other publication** |
| --- | --- | --- | --- | --- | --- | --- | --- | --- | --- | --- | --- | --- |
| 1-1 | 53/Female | FGFR2 EX17-BICC1 EX3 | 2.8 | MF | CLC | 0 | 0 | 0 | Ⅰ | Medium | NA | Pu et al[18] |
| 1-2 | 62/Male | FGFR2 EX17-BICC1 EX3 | 7.5 | MF | CLC | 0 | 0 | 1+ | Ⅰ | Medium | NA | Pu et al[18] |
| 1-3 | 53/Male | FGFR2 EX17-BICC1 EX3 | 6.5 | MF | SD | 0 | 0 | 1+ | Ⅲ | Medium | NA |  |
| 1-4 | 64/Female | FGFR2 EX17-BICC1 EX3 | 1.5 | MF | CLC | 0 | 0 | 1+ | Ⅰ | Low | alive |  |
| 1-5 | 35/Male | FGFR2 EX17-BICC1 EX3 | 4.0 | MF | CLC | 0 | 0 | 1+ | Ⅰ | Medium | NA |  |
| 1-6 | 60/Male | FGFR2 EX17-BICC1 EX18 | 11.5 | MF | CLC | 0 | 2+ | 3+ | Ⅰ | Medium | alive | Pu et al[18] |
| 1-7 | 53/Female | FGFR2 EX17-BICC1 EX18 | 5.5 | MF | CLC | 0 | 1+ | 0 | Ⅱ | Medium | 6months/dead |  |
| 1-8 | 56/Female | FGFR2 EX17-BICC1 EX16 | 6.0 | MF | CLC | 0 | 2+ | 2+ | Ⅰ | Low | 47months/dead |  |
| 1-9 | 48/Male | BICC1 EX3-FGFR2 EX10 | 7.0 | MF | CLC | 0 | 0 | 3+ | Ⅰ | Medium | NA | Pu et al[18] |
| 1-10 | 62/Female | BICC1 EX2-FGFR2 EX18 | 16.0 | MF | SD | 0 | 0 | 1+ | Ⅱ | Low | NA | Pu et al[18] |
| 1-11 | 62/Male | BICC1 EX17-FGFR2 EX18 | 4.0 | MF | CLC | 0 | 0 | 1+ | Ⅱ | Medium | NA | Pu et al[18] |
| 1-12 | 56/Male | FGFR2 EX17-MCU EX2 | 16.0 | MF | CLC | 0 | 0 | 1+ | Ⅲ | Medium | 12month/dead | Pu et al[18] |
| 1-13 | 65/Male | FGFR2 EX17-AHCYL1 EX2 | 5.5 | MF | CLC | 0 | 1+ | - | Ⅰ | Medium | NA |  |
| 1-14 | 52/Male | FGFR2 EX17-UTRN EX51 | 3.7 | MF | CLC | 0 | 1+ | 3+ | Ⅰ | Low | NA |  |
| 1-15 | 55/Male | FGFR2 EX17-RNF41 EX4 | 7.0 | MF | SD | 0 | 0 | 0 | Ⅱ | Medium | NA |  |
| 1-16 | 51/Female | FGFR2 EX17-PKD2L1 EX10 | 7.5 | MF | SD | 0 | 0 | 1+ | Ⅱ | Medium | alive |  |
| 1-17 | 50/Female | FGFR2 EX17-TACC2 EX17 | 7.0 | IG | LD | 0 | 2+ | 1+ | Ⅰ | High | alive |  |
| 1-18 | 61/Male | FGFR2EX17-ATE1 intergenic | 3.8 | MF | AS | 0 | 0 | 1+ | Ⅲ | Medium | alive |  |
| 1-19 | 55/Female | FGFR2 EX17-DDX21 EX2 | 9.0 | MF | CLC | 1+ | 0 | 0 | Ⅲ | Medium | 11 month/dead |  |
| 1-20 | 51/Female | FGFR2 EX17-TFEC EX3 | 6.0 | MF | CLC | 0 | 0 | 3+ | Ⅱ | Medium | alive |  |
| 1-21 | 41/Female | FGFR2 EX17-CCDC6 EX4 | 2.1 | MF | CLC | 0 | 0 | 3+ | Ⅰ | Low | NA |  |
| 1-22 | 73/Female | FGFR2 EX17-SORBS1 EX19 | 6.0 | MF | CLC | 0 | 0 | 1+ | Ⅰ | Medium | NA |  |
| 1-23 | 60/Female | FGFR2 EX17-AFF4 EX2 | 4.5 | MF | CLC | 0 | 0 | 1+ | Ⅰ | Low | NA | Pu et al[18] |
| 1-24 | 50/Male | FGFR2 EX17-PIBF1 EX6 | 3.0 | MF | CLC | 0 | 2+ | 3+ | Ⅰ | Low | 48months/dead | Pu et al[18] |
| 1-25  1-26  1-27  1-28  1-29  1-30 | 58/Male  73/Female  50/Male  43/Male  61/Male  51/Male | LSM6 intergenic-FGFR2 EX18  FGFR2 EX17-CTNNA3 EX14  NA  NA  NA  NA | 4.0  6.5  3.0  9.5  5.5  4.5 | MF  MF  MF  MF  MF  MF | CLC  SD  CLC  CLC  CLC  CLC | 0  0  0  0  0  0 | 1+  0  0  0  0  0 | 0  0  1+  1+  0  1+ | Ⅲ  Ⅲ  Ⅰ  Ⅱ  Ⅰ  Ⅰ | Low  Low  Low  Medium  Medium  Low | alive  NA  alive  NA  NA  NA |  |

Chr: chromosome; RNA: RNA-based NGS; DNA: DNA-based NGS; FISH: fluorescent in situ hybridization; R: red; G: green; MF: mass-forming; IG: intraductal growth; ASC: adenosquamous carcinoma; SD: small duct; CLC: cholangiolocarcinoma; IT: intraductal tumor; NA: not available

Of the 17 FGFR2 translocations revealed by the multiprompt approaches, only 3 showed disagreements among the 3 different approaches.RNA-seq and genomic sequencing showed that Case 17 is a fusion between Exon17 of FGFR2 and Exon17 of TACC2.Since these two genes are both on chromosome 10 and are separated less than 340 kb apart, this fusion was beyond the resolution of our current FISH method. FISH picked up case 18 as a translocation, and RNAseq revealed that it is a fusion of FGFR2 Exon-17 to (intronic sequence) ATE1. Genomic DNA sequencing also failed to identify this fusion, likely because of the proximity of conjoining exonic and intronic sequences. The DNA-seq results likely disagreed with the RNA-seq and FISH results in case 26 for the same reason. Of the 13 translocations uncovered by FISH alone, 9 were confirmed and described previously^18^.The 30 FGFR2 translocations that we uncovered in this cohort of 474 ICCs represent an incidence of 6.33%, in agreement with published assessment for the Chinese population but lower than those of the Japanese andwestern populations^13,19-21^.Our experience indicates that FISH is a reliable and effective method as a first-line screening for gene rearrangement of FGFR2 or any other genes for that matter. The FISH approach also wields the benefit of yielding an unambiguous break-apart signal that is straightforward to interpret, so it is very easy for clinicians or pathologists to use. However, if FISH returns a negative result, RNA-seq should be selected as the method of choice for its definitive outcome, if not the ease of use.

**Table S2** Clinicopathologic, molecular Features and Follow-up Information in Patients with FGFR2 mutation

| Case | Age/Gender | Mutation site | Tumor size  (cm) | Gross classification | Histological classification | Tumor Grade | Tumor differentiation | Follow-up |
| --- | --- | --- | --- | --- | --- | --- | --- | --- |
| 2-1 | 66/Female | p.C382R (c.1144T>C) | 7.5 | MF | SD | Ⅱ | Medium | alive |
| 2-2 | 64/Female | p.P253R (c.758C>G)  p.I548Wfs*8(c.1642_1670delATAAATCTTCTTGGAGCCTGCACACAGGA) | 7.0 | MF+PI | LD | Ⅲ | High | 3 months/dead |
| 2-3 | 64/Female | p.N631_M640del(c.1890_1919delAAATGTTTTGGTAACAGAAAACAATGTGAT) | 7.0 | MF+PI | LD | Ⅲ | High | NA |
| 2-4 | 43/ Female | p.R203C(c.607C>T) | 3.5 | MF | SD | Ⅰ | Medium | alive |

MF: mass-forming; PI: peritubular infiltration; LD: large duct; SD: small duct; NA: not available

**Table S3 Clinicopathologic Features between FGFR2 fusion/translocation positive and negative cases**

| **No. Patients** | **FGFR2**  **translocation**  **(N=30)** | **FGFR2non-translication**  **(N=444)** | ***p* value** |
| --- | --- | --- | --- |
| Average age (year)  Male/Female | 55.50（35-73）  17/13 | 58.33（34-81）  245/199 | 0.165  1.000 |
| HBV infection(+/-)  Cholangitis(+/-)  Fatty liver(+/-)  Clonorchiasis(+/-) | 16/14  22/8  12/18  3/27 | 266/178  243/201  166/278  22/422 | 0.565  0.057  0.846  0.205 |
| Tumor numbers(n)  Tumormaximum dimension (cm) | 3.15(1-10)  6.52(2.8-11.5) | 2.64(1-11)  5.85(0.5-16) | 0.365  0.312 |
| Gross classification  MF type  non-MF type | 29  1 | 278  166 | **0.000*** |
| Histological classification  LD  SD  CLC  others | 1  5  23  1 | 186  205  49  4 | **0.000*** |
| Differentiation (L/M/H) | 11/18/1 | 196/236/12 | 0.724 |
| G (0/1/2/3/4)  S (0/1/2/3/4)  T(I/II/III/IV)  Stage(I/II/III/IV) | 12/15/1/1/1  14/9/12/4/1  17/7/6/0  15/13/1/1 | 143/194/58/40/9  76/155/145/46/22  161/164/100/19  142/183/78/41 | 0.381  0.074  0.116  0.063 |
